# Anaerobic Thermophiles

**DOI:** 10.3390/life4010077

**Published:** 2014-02-26

**Authors:** Francesco Canganella, Juergen Wiegel

**Affiliations:** 1 Department for Innovation in Biological, Agrofood, and Forest Systems, University of Tuscia, via C. de Lellis, Viterbo 01100, Italy; 2 Department of Microbiology, University of Athens (GA), Athens 10679, USA; E-Mail: juergenwiegel@gmail.com

**Keywords:** anaerobic thermophiles, thermal ecosystems, extremophiles, deep-sea, taxonomy, biotechnology

## Abstract

The term “extremophile” was introduced to describe any organism capable of living and growing under extreme conditions. With the further development of studies on microbial ecology and taxonomy, a variety of “extreme” environments have been found and an increasing number of extremophiles are being described. Extremophiles have also been investigated as far as regarding the search for life on other planets and even evaluating the hypothesis that life on Earth originally came from space. The first extreme environments to be largely investigated were those characterized by elevated temperatures. The naturally “hot environments” on Earth range from solar heated surface soils and water with temperatures up to 65 °C, subterranean sites such as oil reserves and terrestrial geothermal with temperatures ranging from slightly above ambient to above 100 °C, to submarine hydrothermal systems with temperatures exceeding 300 °C. There are also human-made environments with elevated temperatures such as compost piles, slag heaps, industrial processes and water heaters. Thermophilic anaerobic microorganisms have been known for a long time, but scientists have often resisted the belief that some organisms do not only survive at high temperatures, but actually thrive under those hot conditions. They are perhaps one of the most interesting varieties of extremophilic organisms. These microorganisms can thrive at temperatures over 50 °C and, based on their optimal temperature, anaerobic thermophiles can be subdivided into three main groups: thermophiles with an optimal temperature between 50 °C and 64 °C and a maximum at 70 °C, extreme thermophiles with an optimal temperature between 65 °C and 80 °C, and finally hyperthermophiles with an optimal temperature above 80 °C and a maximum above 90 °C. The finding of novel extremely thermophilic and hyperthermophilic anaerobic bacteria in recent years, and the fact that a large fraction of them belong to the *Archaea* has definitely made this area of investigation more exciting. Particularly fascinating are their structural and physiological features allowing them to withstand extremely selective environmental conditions. These properties are often due to specific biomolecules (DNA, lipids, enzymes, osmolites, *etc.*) that have been studied for years as novel sources for biotechnological applications. In some cases (DNA-polymerase, thermostable enzymes), the search and applications successful exceeded preliminary expectations, but certainly further exploitations are still needed.

## 1. Introduction

**Figure 1:**
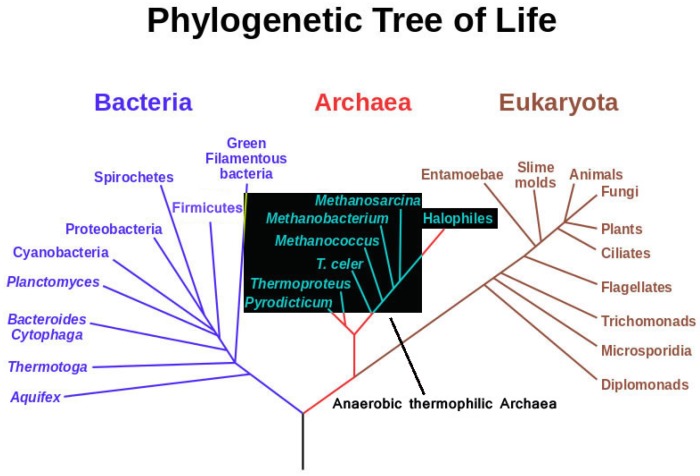
Phylogenetic tree highlighting possible evolutionary relatedness of anaerobic thermophilic Archaea (modified from Eric Gaba, NASA Astrobiology Institute 2006).

Among anaerobic and thermophilic microorganisms, anaerobic thermophilic Archaea are certainly the most “extreme” in terms of inhabited ecosystems. They represent the deepest, least evolved branches of the universal phylogenetic tree (Figure 1). They often use substrates, which are thought to have been dominant in the primordial terrestrial makeup, indicating that they could have been the first living forms on this planet [1,2,3,4,5,6]. Studies into how they manage thermostability at the protein and membrane structural level have elucidated many traits of protein, membrane and nucleic acid structure; however, there is not yet a full understanding of the principles of thermostability [7,8,9,10,11]. The development of better genetic tools for the use of these organisms is the key for more practical applications in the future [12,13,14].

Although the first forms of life no longer exist, natural thermal environments are still abundant on Earth and some have properties similar to those environments in which life possibly first began. Many of these environments are characteristically anaerobic or have low levels of oxygen. The anaerobic feature can stem from a number of factors: remoteness of the environment from the atmosphere, low solubility of oxygen in water at elevated temperatures, hypersalinity, inputs of reducing gasses such as H_2_S, or the consumption of oxygen by aerobic microorganisms on or near the water surface.

**Figure 2:**
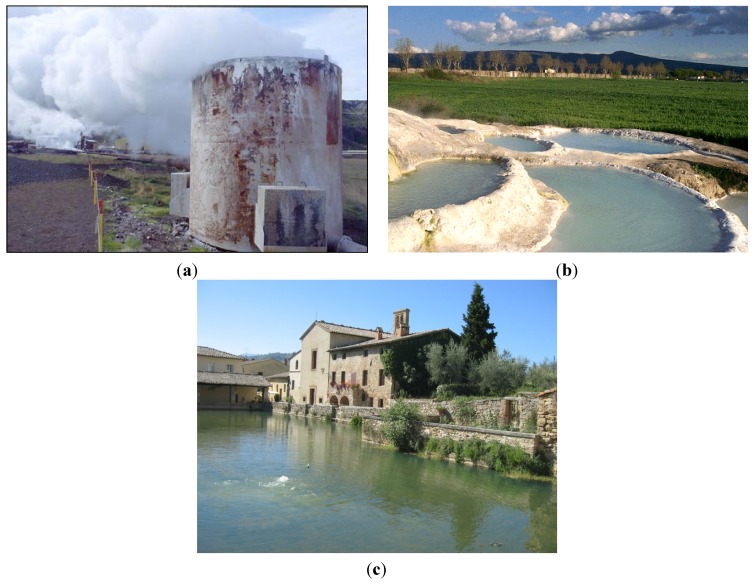
Some environments where anaerobic thermophiles can be isolated: (**a**) A power plant in Iceland; (**b**) Terrestrial hot springs at Viterbo (Italy); (**c**) The hot pool of Bagno Vignoni (Italy).

**Figure 3:**
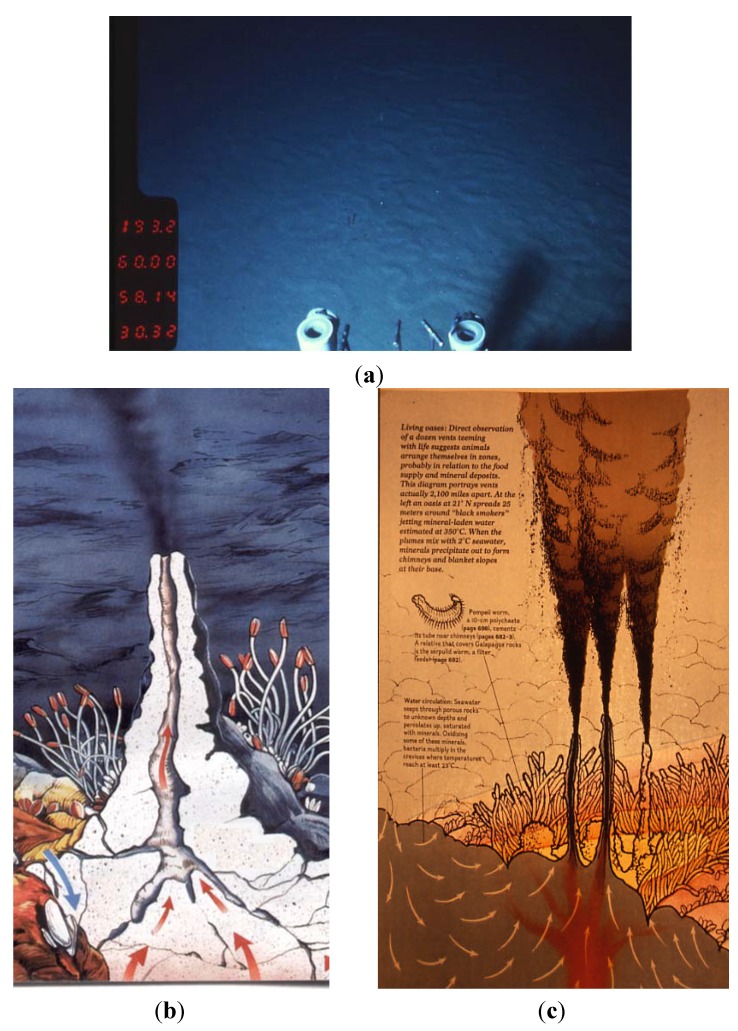
Deep-sea hot ecosystems: (**a**) Hot sediment at the Guaymas Basin; (**b**,**c**) Drawings of black smokers located at a deep-sea hydrothermal vent area (courtesy of Focus Magazine and Jack Jones, respectively).

Natural environments for anaerobic thermophiles range from terrestrial volcanic sites (including solfatara fields) with temperatures slightly above ambient temperature, to submarine hydrothermal systems (sediments, submarine volcanoes, fumaroles and vents) with temperatures exceeding 300 °C, subterranean sites such as oil reservoirs, and solar heated surface soils with temperatures up to 65 °C (Figure 2 and Figure 3). There are also human-made hot environments such as compost piles (usually around 60–70 °C but as high as 100 °C) slag heaps, industrial processes and water heaters [15].

Oil reservoirs, mines, and geothermal aquifers are examples of subsurface environments that thermophiles populate. Extreme thermophilic bacterial species of the genera *Geotoga* and *Petrotoga* (family *Thermotogaceae*) have so far only been found in deep subsurface oil reservoirs; on this basis, it has been proposed that these taxa represent typical indigenous *Bacteria* of this particular ecosystem. However, lately Thermotogales sequences have been found in mesobiotic environments [16] and novel species have been described [17]. Geothermal aquifers, such as the Great Artesian Basin of Australia, are considered to be markedly different from volcanically related hot springs in that they have low flow rates and long recharge times (around 1000 years) that affect the microbial populations therein. Besides natural thermal environments, thermophilic anaerobes are also found within anthropogenically heated environments, including coal refuse piles and compost heaps, and nuclear power plant effluent channels which contain not only spore-forming species, but also vegetative and active cells including *Bacteria* and *Archaea*.

Many environments are also temporarily hot, adaptation to which may be the reason some thermophiles are very fast-growing. Among the geothermally heated habitats are the alkaline, mainly carbonate-containing hot springs around a neutral pH, and acidic areas including some mud-holes. Most of the acidic high-temperature habitats contain elemental sulfur and metal sulfides and most isolates from these areas metabolize sulfur by either anaerobic respiration or fermentation. Ocean depths are under extreme pressures from the weight of the water column, and thus most anaerobic and thermophilic bacteria from these areas are piezotolerant, some are truly piezophilic, others such as *Pyrococcus* spp., *Thermococcus* spp., and *M. kandleri* show extensions of T_max_ under increased pressure [18,19,20,21,22,23] and all are at least halotolerant [24], and those isolated from solfataras generally acidophilic. Alike most described species of obligately aerobic thermophilic *Archaea* that are acidophilic, anaerobic thermophilic bacteria are generally unable to grow at acidic pH with some exemptions such as representatives of genera *Stygiolobus*, *Acidilobus*, and *Caldisphaera* [25,26,27]. On the other hand many anaerobic bacteria and some *Archaea* are capable of growing at an alkaline pH [28]. The anaerobic alkalithermophilic bacteria thus form an interesting group to study, and their relationships between temperature optimum and pH optimum for growth have been extensively investigated. This adaptability to high pH environments involves both cellular and biomolecular peculiar traits that are currently under investigation, particularly to exploit their potential biotechnological applications.

Among extreme environments, the deep sea is in general cold, but it is known to show areas of superheated water and widespread still-hot volcanic ocean crust beneath the flanks of the mid-ocean ridge and other rock structures, as well as geothermally heated shallower ocean waters.

A large group of anaerobic and thermophilic microorganisms have been isolated and studied from the deep-sea, particularly at both hydrothermal vents and sub-seafloor sites, either for their physiological properties or for their potential applications [29,30,31,32,33,34]. Representative deep-sea environments, if not in terms of geographical extension but certainly as the most spectacular, are the deep-sea hydrothermal vents. The highly dense and biologically diverse communities in the immediate vicinity of hydrothermal vent flows are in stark contrast to the surrounding bare seafloor. They comprise organisms with distinct metabolisms based on chemosynthesis and growth rates comparable to those from shallow water tropical environments, which have been rich sources of biologically active natural products. Fundamental discoveries in this regard will be accelerated by new cost-effective technologies in deep-sea research and more advanced molecular techniques.

Taxonomical and phylogenetic investigations have always been the main focus concerning research on deep-sea anaerobic thermophiles. Diversity and richness of deep-sea hydrothermal environments were particularly examined and shown to be as high as those in soil. As a matter of fact, sediments from deep-sea floors have always been great sources of novel bacterial isolates and recently new genera as well as species are being described from different sites in the ocean depths [35,36,37].

As far as concerns the diversity of sub-seafloor microorganisms, a “meta-enzyme approach” has been proposed as an ecological enzymatic method to explore the potential functions of microbial communities in extreme environments such as the deep marine habitats [38]. Detectable enzyme activities were used to predict the existence of a sizable population of viable anaerobic microorganisms even in deep sub-seafloor habitats. Moreover many microbial isolates produced a variety of extra-cellular enzymes such as proteases, amylases, lipases, chitinases, phosphatases, and deoxyribonucleases, giving them a great potential in terms of biotechnological applications.

A main topic in ecology and population dynamics of deep-sea anaerobic thermophiles is their colonization and distribution patterns along and around hydrothermal vent deposits. An approach based on the deployment of thermocouple arrays on two deep-sea hydrothermal vents at Guaymas Basin was adopted by Pagé *et al.* [39]. This aimed to measure *in situ* temperatures at which microorganisms colonize the associated mineral deposits. Spatial differences in archaeal diversity were observed in all deposits in relationship to *in situ* temperature. This study was the first direct assessment of *in situ* conditions experienced by microorganisms inhabiting actively forming hydrothermal deposits at different stages of structure development.

## 2. Growth Conditions

Microorganisms that grow optimally at elevated temperatures above 50 °C and can not use oxygen as terminal electron acceptor during electron transport phosphorylation are described as thermophilic anaerobes. They are of interest from basic and applied scientific perspectives and are studied to understand how life can thrive in environments previously considered inhospitable to life. Such environments include volcanic solfataras and hot springs high in sulfur and toxic metals, as well as abyssal hydrothermal vents with extremely high pressure and temperatures far above 100 °C [40].

Isolated species of thermophilic anaerobes include peculiar forms: for example, cells of the alkalithermophile *Clostridium paradoxum* become highly motile when sporulating, and *Moorella thermacetica*-like strains have exceptionally heat-resistant spores with D_10_ times of nearly 2 h at 121 °C. Also, *Pyrolobus fumarii* grows optimally at 106 °C, and the record-holder *Methanopyrus* *kandleri*-like strain grows at 122 °C under increased pressure [18]. *Thermobrachium celere* strains have doubling times of about 10 min while growing above pH 9.0 and above 55 °C [41] and the polyextremophilic *Natranaerobius* isolates simultaneously grow optimally up to 69 °C and above pH 9.5, and at a salt concentration above 4 M Na^+^. They may be considered the most extremophile as they withstand the combination of multiple stressors. It will be of interest to evaluate whether those boundaries can be further extended by isolating other polyextremophiles [42,43,44].

The analyses of the biodiversity and patterns of biodiversity within thermal environments is an area of active research that continually expands as technology allows for novel approaches and more detailed analyses. Additionally, their thermostable enzymes, among other characteristics, make thermophilic anaerobes of significant interest for their biotechnological potential.

Contrary to any expectation, thermophilic anaerobes have also been isolated from mesobiotic and even psychrobiotic environments: two *Thermosediminibacter* species were isolated from ocean sediments of the Peru Margin at temperatures at or below 12 °C [45], uncharacterized *Thermoanaerobacter* species have been isolated from melted snow from Antarctica (unpublished results), alkalithermophiles have been isolated from many river sediments and wet meadows, and *Methanothermobacter thermoautotrophicus* and other thermophilic methanogens and chemolithoautotrophic acetogens can readily be found in lake sediments and rivers, streams, and ponds. Possible reasons for the presence of thermophilic anaerobes in environments where they were thought not to grow, considering their physiological properties, include (a) that the microorganisms are present but do not grow in these environments although they are able to carry out maintenance metabolism (e.g., as shown for *M. thermoautotrophicum* that is able to form methane at temperatures as low as 16 °C, although it is not able to multiply at temperatures below 22 °C (Wiegel unpl. results); (b) that they disperse only transiently from other thermal environments; (c) that they survive and multiply by taking advantage of temporary thermal piconiches that become available when proteinaceous biomass is degraded. The latter notion is further substantiated by observations that strains of *Calaromator* (Bas. *Thermobrachium) celer* isolated from mesobiotic environments show very short doubling times (between 10 min and 20 min), whereas the strains of the same species isolated from hot springs—which resemble a more constant thermobiotic environment—have doubling times of above 30 min (Wiegel, unpublished results) and also that the moderate thermophiles *C. paradoxum-* and the nonsporulating *C. thermophilus*-like cells are present in mesobiotic sewage sludge (<30 °C) at 1000 CFU/mL sludge [46]. However, so far, no direct molecular methods have been used to explain the growth of these taxa *in vivo*.

Thermophilic anaerobes in pure culture are characterized by a polyphasic approach, in which phenotypic and genotypic/phylogenetic properties are examined. Phenotypic characteristics of particular interest for this discussion include oxygen relationships and metabolic properties, such as energy production and carbon assimilation. Group-defining properties for extremophiles (also called marginal data set), such as temperature growth range (e.g., T_min_, T_opt_, and T_max_) and pH growth range (e.g., pH_min_, pH_opt_, and pH_max_), are particularly important. These values should be determined by measuring the doubling times over the range for growth and specifically noting where growth was obtained and where growth was not obtained. For other thermophilic extremophiles besides thermophilic alkali-/acido-thermophiles, the range and optima for their other characteristic properties, such as salt (halophiles) pressure (piezophiles), substrate concentration (oligophiles, osmophiles) and tolerance to metals or solvents are important when considering thermophilic anaerobes from habitats such as sun heated hypersaline lakes and deep-sea hydrothermal regions, deep oil wells/oil storage tanks, or heavily contaminated thermobiotic sites. Although genotypic characteristics such as G + C mol% of the genomic DNA and DNA-DNA relatedness between strains have been studied since the 1960s, in the past 20 years analysis of the 16S rRNA gene sequence (frequently backed up by DNA: DNA hybridization studies for taxa with 16S rRNA sequence similarity above 97%) has become standard, and the analysis of house keeping genes and more recently, the whole-genome sequencing of prokaryotes has become increasingly more common. As we enter deeper and deeper into the genomic era, genome sequencing will certainly become an essential part of the characterization and differentiation of novel taxa exceeding the importance of the 16S rRNA gene sequence analysis used today. In Table 1, classified thermophilic anaerobes with available sequenced genomes are reported, however, for most recent information, the reader should refer to the National Center for Biotechnology Information Taxonomy Database [47].

**Table 1:** Most representative thermophilic anaerobes with official nomenclature and genome sequenced.

Species	O_2_-relationship and metabolism	Temperature range (optimum)	pH range (optimum)	Originally isolated from
***Bacteria*; *Proteobacteria*; *Gammaproteobacteria*; *Chromatiales*; *Chromatiaceae*; **				
**Genus: *Thermochromatium***				
*Thermochromatium* *tepidum*	AN	34–57 (48–50)	(7)	Mammoth Hot Spring, Yellowstone National Park, USA
	PA/PH			
***Bacteria*; *Bacteroidetes/Chlorobi* group; *Bacteroidetes*; *Bacteroidetes*; *Bacteroidales*; *Bacteroidaceae*; **				
**Genera: *Acetomicrobium*, *Anaerophaga***				
*Anaerophaga* sp. strain HPS1	NR	NR	NR	Offshore hot spring sediment, China
***Bacteria*; *Spirochaetes*; *Spirochaetes*; *Spirochaetales*; *Spirochaetaceae*; **				
**Genus: *Spirochaeta*, *Exilispira***				
*Spirochaeta* *thermophila*	AN	40–73 (66–68)	5.9–7.7 (7.5)	Marine hot spring on the beach of an island of Kamchatka, also from a hot spring on Raoul Island, New Zealand
	COH			
***Bacteria*; *Firmicutes*; *Clostridia*; *Halanaerobiales*; *Halanaerobiaceae*;**				
**Genus: *Halothermothrix***				
*Halothermothrix* *orenii*	AN	45–68 (60)	5.5–8.2 (6.5–7)	Chott El Guettar hypersaline lake, Tunisia
	COH			
***Bacteria*; *Firmicutes*; *Clostridia*; *Natranaerobiales*; *Natranaerobiaceae*;**				
**Genus: *Natranaerobius***				
*Natranaerobius thermophilus*	AN	35–56 (53)	8.5–10.6 (9.5)	Sediment of alkaline, hypersaline lakes of the Wadi An Natrun
	COH			
***Bacteria*; *Firmicutes*; *Clostridia*; *Thermoanaerobacteriales*; *Thermoanaerobacteriaceae*; *Syntrophomonadaceae*;**				
**Genera: *Coprothermobacter*, *Gelria*, *Moorella*, *Thermacetogenium*, *Mahella*, *Thermoanaerobacterium*, *Thermoanaerobacter*, *Thermosediminibacter*, *Caldanaerobacter*, *Thermovenabulum*, *Tepidanaerobacter*, *Ammonifex*, *Thermanaeromonas*, *Thermhydrogenium*, *Caldanaerovirga*, *Fervidicola*, *Caldanaerobius***				
*Coprothermobacter* *proteolyticus*	AN	35–70 (63)	5–8.5 (7.5)	Thermophilic digester fermenting tannery wastes and cattle manure
	COH			
*Moorella thermoacetica*	AN	45–65 (55–60)	NR	Horse manure
	COH			
*Thermoanaerobacter* *ethanolicus*	AN	37–78 (69])	4.4–9.9 (5.8–8.5)	Hot springs, Yellowstone National Park, USA
	COH			
*Thermoanaerobacter pseudoethanolicus*	AN	(65)	NR	Hot Spring, Yellowstone National Park, USA
	COH			
*Caldanaerobacter subterraneus* subsp*. tengcongensis*	AN	50–80 (75)	5.5–9 9 (7–7.5)	Hot spring, Tengcong, China
	COH			
*Ammonifex* *degensii*	AN	57–77 (70)	5–8 (7.5)	Kawah Candradimuka crater, Dieng Plateau, Java, Indonesia
	F-CLA			
***Bacteria*; *Firmicutes*; *Clostridia*; *Clostridiales*; *Acidaminococcaceae*; **				
**Genus: *Thermosinus***				
*Thermosinus* *carboxydivorans*	AN	40–68 (60)	6.5–7.6 (6.8–7)	Norris Basin hot spring, Yellowstone National Park, USA
	CLA			
***Bacteria*; *Firmicutes*; *Clostridia*; *Clostridiales*; *Peptococcaceae*;**				
**Genera: *Desulfotomaculum*, *Pelotomaculum*, *Carboxydothermus*, *Thermincola***				
*Pelotomaculum* *thermopropionicum*	AN	45–65 (55)	6.7–7.5 (7)	Thermophilic upflow anaerobic sludge blanket reactor
	COH			
*Carboxydothermus* *hydrogenoformans*	AN	40–78 (70-72)	6.4–7.7 (6.8–7)	Freshwater hydrothermal springs, Kunashir Island, Kamchatka, Russia
	CLA			
***Bacteria*; *Firmicutes*; *Clostridia*; *Clostridiales*; *Syntrophomonadaceae*;**				
**Genera: *Anaerobaculum*, *Syntrophothermus*, *Thermanaerovibrio*, *Carboxydocella*, *Anaerobranca*, *Thermosyntropha*, *Caldicellulosiruptor***				
*Caldicellulosiruptor* *lactoaceticus*	AN	50–78 (68)	5.8–8.2 (7)	Hveragerði alkaline hot spring, Iceland
	COH			
*Caldicellulosiruptor* *owensensis*	AN	50–80 (75)	5.5–9 (7.5)	Freshwater pond within the dry Owens Lake bed, California, USA
	COH			
*Caldicellulosiruptor* *kristjanssonii*	AN	45–82 (78)	5.8–8 (7)	Hot spring, Iceland
	COH			
*Caldicellulosiruptor* *saccharolyticus*	AN	45–80 (70)	5.5–8.0 (7.0)	Geothermal spring, Taupo, New Zealand
	COH			
*Caldicellulosiruptor bescii*	AN			
	COH			
*Caldicellulosiruptor kronotskyensis*	AN	(70)	(7)	Hot spring, Kamchatka, Russia
	COH			
*Caldicellulosiruptor hydrothermalis*	AN	(65)	(7)	Hot spring, Kamchatka, Russia
	COH			
***Bacteria*; *Firmicutes*; *Clostridia*; *Clostridiales*; *Heliobacteriaceae*; **				
**Genus: *Heliobacterium***				
*Heliobacterium* *modesticaldum*	AN	25–56 (52)	(6–7)	Iceland, Yellowstone National Park, USA
	PH & COH			
***Bacteria*; *Firmicutes*; *Clostridia*; *Clostridiales*; *Clostridiaceae*; *Caldicoprobacteraceae*; *Veillonellaceae*;**				
**Genera: *Alkaliphilus*, *Clostridium*, *Tepidibacter*, *Caloramator*, *Garciella*, *Caminicella*, *Caloranaerobacter*, *Thermobrachium*, *Thermohalobacter*, *Tepidimicrobium*, *Fervidicella*, *Caldicoprobacter*, *Sporolituus*, *Thermotalea*, *Lutispora***				
*Clostridium thermocellum*	AN	28–69 (60)	(6.1–7.5)	Louisiana cotton bale and Compost heap
	COH			
*Clostridium* *stercorarium* subsp. *stercorarium*	AN	(65)	(7.3)	Compost heap
	COH			
***Bacteria*; *Firmicutes*; *Bacilli*; *Bacillales*; *Bacillaceae*;**				
**Genera: *Anoxybacillus*, *Bacillus*, *Geobacillus*, *Vulcanibacillus***				
*Anoxybacillus* *flavithermus*	FAE	30–72 (60–65)	5.5–9 (7)	A hot spring, New Zealand
	COH			
*Geobacillus thermodenitrificans*	FAE	45–70	6–8	Sugar beet juice from extraction installations; Austria
	COH			
*Geobacillus thermoleovorans*	FAE	35–78 (55–65)	(6.2–6.8)	Soil near hot water effluent, Bethlehem, PA, USA
	COH			
*Geobacillus thermoglucosidiasus*	FAN	40–70 (60)	6–9 (7)	Japan soil
	COH			
***Bacteria*; *Proteobacteria*; delta/epsilon subdivisions; *Deltaproteobacteria*; *Desulfurellales*; *Desulfurellaceae*;**				
**Genera: *Desulfurella*, *Hippea***				
*Hippea* *maritima*	AN	40–65 (52–54)	5.4–6.5 (5.8–6.2)	Shallow water hot vents, Bay of Plenty, New Zealand and Matupi Harbour, Papua New Guinea
	COH			
***Bacteria*; *Proteobacteria*; delta/epsilon subdivisions; *Epsilonproteobacteria*; *Nautiliales*; *Nautiliaceae*;**				
**Genera: *Nautilia*, *Lebetimonas*, *Caminibacter***				
*Caminibacter* *mediatlanticus*	AN	45–70 (55)	4.5–7.5 (5.5)	“Rainbow” deep-sea vent field, Mid-Atlantic Ridge
	CLA			
***Bacteria*; *Deferribacteres*; *Deferribacteres*; *Deferribacterales*; *Deferribacteraceae*;**				
**Genera: *Deferribacter*; *Flexistipes* also, *Caldithrix* (unclassified *Deferribacteres*), *Calditerrivibrio***				
*Deferribacter desulfuricans*	AN	40–70 (60–65)	5.0–7.5 (6.5)	From a black smoker vent from the hydrothermal fileds at the Suiyo Seamount in the Izu-Bonin Arc, Japan
	COH			
***Bacteria*; *Thermodesulfobacteria*; *Thermodesulfobacteria*; *Thermodesulfobacteriales*; *Thermodesulfobacteriaceae*; **				
**Genera: *Thermodesulfatator*, *Thermodesulfobacterium*, *Caldimicrobium*, *Thermosulfidibacter***				
*Thermodesulfatator* *indicus*	AN	55–80 (70)	6–6.7 (6.25)	The Kairei deep-sea hydrothermal vent field, Central Indian Ridge
	CLA			
*Thermodesulfobacterium commune*	AN	50–85 (70)	6.0–8.0	Ink Pot Spring, Yellowstone National Park, USA
	COH			
***Bacteria*; *Nitrospirae*; *Nitrospira*; *Nitrospirales*; *Nitrospiraceae*; **				
**Genus: *Thermodesulfovibrio***				
*Thermodesulfovibrio* *yellowstonii*	AN	40–70 (65)	(6.8–7)	Thermal vent, Yellowstone National Park, USA
	COH			
***Bacteria*; *Dictyoglomi*; *Dictyoglomi*; *Dictyoglomales*; *Dictyoglomaceae*; **				
**Genus: *Dictyoglomus***				
*Dictyoglomus* *thermophilum*	AN	50–80 (73–78)	5.9–8.3 (7)	Hot spring, Kumamoto Prefecture, Japan
	COH			
***Bacteria*; *Chloroflexi*; *Chloroflexi*; *Chloroflexales*; *Chloroflexaceae*; **				
**Genera: *Roseiflexus*, *Chloroflexus*, *Heliothrix***				
*Roseiflexus* *castenholzii*	FAE	45–55 (50)	7–9 (7.5–8)	Hot spring, Nakabusa, Japan
	PH (anaerobic)			
*Chloroflexus aggregans*	FAE	(50–60)	7.0–9.0	Hot spring of the Okukinu Meotobuchi hot spring in Tochigi Perfecture, Japan
	PH (anaerobic)			
*Chloroflexus aurantiacus*	FAE	(52–60)	(8)	Hot spring in the canyon at Sokokura, Hakone district, Japan
	PH (anaerobic)			
*Heliothrix oregonensis*	FAE	(40–55)	NR	Hot spring near Warm Springs River, Oregon, USA
	PH			
***Bacteria*; *Chloroflexi*; *Thermomicrobia*; *Thermomicrobiales*; *Thermomicrobiaceae*; **				
**Genus: *Thermomicrobium***				
*Thermomicrobium roseum*	AN	(70–75)	6–9.4 (8.2–8.5)	Hot spring, Yellowstone National Park, USA
	COH			
***Bacteria*; *Aquificae*; *Aquificae*; *Aquificales*; *Aquificaceae*; **				
**Genera: *Hydrogenivirga*, *Aquifex*, *Desulfurobacterium* (unclassified *Aquificales*), *Balnearium* (unclassified *Aquificales*), *Thermovibrio* (unclassified *Aquificales*)**				
*Desulfurobacterium* *thermolithotrophum*	AN	40–75 (70)	4.4–7.5 (6)	“Snake Pit” vent field, Mid-Atlantic ridge
	CLA			
*Thermovibrio* *ammonificans*	AN	60–80 (75)	5–7 (5.5)	Deep sea hydrothermal vent area, East Pacific Rise
	CLA			
***Bacteria*; *Aquificae*; *Aquificae*; *Aquificales*; *Hydrogenothermaceae*; **				
**Genera: *Hydrogenothermus*, *Sulfurihydrogenibium*, *Persephonella***				
*Sulfurihydrogenibium* *azorense*	FAE	50–73 (68)	5.5–7 (6)	Near the Água do Caldeirão, Furnas, on São Miguel Island, Azores
	CLA			
***Bacteria*; *Thermotogae*; *Thermotogae*; *Thermotogales*; *Thermotogaceae*;**				
**Genera: *Geotoga*, *Marinitoga*, *Petrotoga*, *Thermosipho*, *Thermotoga*, *Fervidobacterium*, *Thermococcoides, Kosmotoga***				
*Marinitoga* *camini*	AN	25–65 (55)	5–9 (7)	Deep sea vent fields, Mid-Atlantic ridge
	COH			
*Petrotoga* *mobilis*	AN	40–65 (58–60)	5.5–8.5 (6.5–7)	Oil reservoir production water from off-shore oil platforms, North Sea
	COH			
*Thermosipho* *melanesiensis*	AN	45–80 (70)	3.5–9.5 (6.5–9.5)	Deep sea hydrothermal area, Lau Basin, southwest Pacific Ocean
	COH			
*Fervidobacterium nodosum*	AN	41–79 (70)	6–8 (7)	Hot spring in New Zealand
	COH			
*Thermotoga* *lettingae*	AN	50–75 (65)	6–8.5 (7)	Thermophilic, sulfate-reducing, slightly saline bioreactor
	COH			
**Thermotoga maritima**	**AN**	**55–90 (80)**	**5.5–9 (6.5)**	**Geothermally heated sea floors, Italy and the Azores**
	**COH**			
**Thermotoga petrophila**	**AN**	**47–88 (80)**	**5.2–9 (7)**	**Production fluid of the Kubiki oil reservoir in Niigata, Japan**
	**COH**			
**Thermotoga neapolitana**	**AN**	**55–90 (80)**	**5.5–9 (7)**	**Shallow submarine hot springs, Lucrino Bay, Naples, Italy**
	**COH**			
***Archaea*; *Crenarchaeota*; *Thermoprotei*; *Desulfurococcales*; *Desulfurococcaceae***				
**Genera: *Acidilobus*, *Staphylothermus*, *Ignicoccus*, *Desulfurococcus Thermosphaera*, *Sulfophobococcus*, *Stetteria*, *Thermodiscus*, **(Also *Ignisphaera* of the ***Ignisphaera*** group)				
*Thermosphaera* *aggregans*	AN	65–90 (85)	5–7 (6.5)	“Obsidian Pool” Yellowstone National Park, USA
	COH			
*Staphylothermus* *marinus*	AN	65–98 (92)	4.5–8.5 (6.5)	Vulcano Island, Italy, also a deep-sea black smoker of the East Pacific Rise
	COH			
***Archaea*; *Crenarchaeota*; *Thermoprotei*; *Desulfurococcales*; *Pyrodictiaceae*;**				
**Genera: *Pyrodictium*, *Hyperthermus*, *Pyrolobus***				
*Hyperthermus* *butylicus*	AN	(95–107)	(7)	Hydrothermally heated flat-sea sediments off the coast of São Miguel Island, Azores
	COH			
***Archaea*; *Crenarchaeota*; *Thermoprotei*; *Thermoproteales*; *Thermofilaceae***				
**Genus: *Thermofilum***				
*Thermofilum* *pendens*	AN	(85–90)	(5)	Icelandic solfataras
	COH			
***Archaea*; *Crenarchaeota*; *Thermoprotei*; *Thermoproteales*; *Thermoproteaceae***				
***Genera*: *Thermoproteus*, *Pyrobaculum*, *Thermocladium*, *Caldvirga***				
*Thermoproteus* *neutrophilus*	AN	(85)	(6.8)	Hot spring, Iceland
	F-CLA			
*Pyrobaculum* *arsenaticum*	AN	68–100 (81)	NR	Hot water pond, Pisciarelli Solfatara, Naples, Italy
	F-CLA			
*Pyrobaculum* *islandicum*	AN	74–102 (100)	5–7 (6)	Boiling solfataras and geothermal waters, Iceland
	F-CLA			
*Pyrobaculum calidifontis*	FAE	75–100 (90-95)	5.5–8.0 (7.0)	Terrestrial hot spring Calamba, Laguna, the Philippines
	COH			
*Pyrobaculum* *aerophilum*	FAE	75–104 (100)	5.8–9 (7)	Boiling marine water hole, Maronti Beach, Ischia, Italy
	F-CLA			
*Caldivirga maquilingensis*	FAE	62–92 (85)	2.3–6.4 (3.7–4.2)	Acidic hot spring in the Philippines
	COH			
***Archaea*; *Euryarchaeota*; *Thermoplasmata*; *Thermoplasmatales*; *Thermoplasmataceae*;**				
**Genus: *Thermoplasma*; *Acidiplasma***				
*Thermoplasma* *acidophilum*	FAE	45–63 (59)	0.5–4 (1–2)	Solfatara fields and self heated coal refuse piles
	COH			
*Thermoplasma* *volcanium*	FAE	33–67 (60)	1–4 (2)	Submarine and continental solfataras at Vulcano Island, Italy; also from Java, Iceland and Yellowstone National Park, USA
	COH			
***Archaea*; *Euryarchaeota*; *Methanococci*; *Methanococcales*; *Methanocaldococcaceae***				
**Genera: *Methanocaldococcus*, *Methanotorris***				
*Methanocaldococcus* *jannaschii*	AN	50–86 (85)	5.2–7.0 (6.0)	“White smoker” chimney on the 20°N East Pacific Rise
	CLA			
*Methanocaldococcus* *vulcanius*	AN	49–89 (80)	5.2–7 (6.5)	Deep-sea vent, 13°N thermal field, East Pacific Rise
	CLA			
***Archaea*; *Euryarchaeota*; *Thermococci*; *Thermococcales*; *Thermococcaceae*;**				
**Genera: *Thermococcus*, *Pyrococcus*, *Palaeococcus***				
*Thermococcus* *barophilus*	AN	48–100 (85)	(7)	“Snakepit” hydrothermal vent region of the Mid-Atlantic ridge
	COH			
*Thermococcus* *gammatolerans*	AN	55–95 (88)	(6)	Guaymas Basin, Gulf of California
	COH			
*Thermococcus kodakarensis*	AN	60–100 (85)	5–9 (6.5)	Solfatara on Kodakara Island, Kagoshima, Japan
	COH			
*Thermococcus* *sibiricus*	AN	40–88 (78)	5.8–9 (7.5)	Samotlor oil reservoir, Western Siberia
	COH			
*Pyrococcus* *furiosus*	AN	70–103 (100)	5–9 (7)	Shallow marine hydrothermal system at Vulcano Island, Italy
	COH			
*Pyrococcus* *horikoshii*	AN	80–102 (98)	5–8 (7)	Hydrothermal fluid samples obtained at the Okinawa Trough vents in the NE Pacific Ocean, at a depth of 1395 m
	COH			
***Archaea*; *Euryarchaeota*; *Archaeoglobi*; *Archaeoglobales*; *Archaeoglobaceae*;**				
**Genera: *Archeoglobus*, *Geoglobus*, *Ferroglobus***				
*Archaeoglobus fulgidus*	AN	64–92 (83)	5.5–7.5	Marine hydrothermal systems at Vulcano island and at Stufe di Nerone, Naples, Italy
	F-CLA			
***Archaea*; *Euryarchaeota*; *Methanopyri*; *Methanopyrales*; *Methanopyraceae*;**				
**Genus: *Methanopyrus***				
*Methanopyrus* *kandleri*	AN	84–110 (98)	5.5–7 (6.5)	Deep-sea sediment from the Guaymas Basin, Gulf of California, and from the shallow marine hydrothermal system of the Kolbeinsey ridge, Iceland
	CLA			
***Archaea*; *Euryarchaeota*; *Methanobacteria*; *Methanobacteriales*; *Methanobacteriaceae*;**				
**Genera: *Methanobacterium*, *Methanothermobacter***				
*Methanothermobacter* *thermautotrophicus*	AN	40–75 (65–70)	6.0–8.8 (7.2–7.6)	Anaerobic sewage sludge digestor
	CLA			
***Archaea*; *Euryarchaeota*; *Methanococci*; *Methanococcales*; *Methanococcaceae*;**				
***Genus*: *Methanothermococcus***				
*Methanothermococcus thermolithotrophicus*	AN	30–70 (65)	6–8 (7)	Heated sea sediments near Naples, Italy
	CLA			
***Archaea*; *Euryarchaeota*; *Methanomicrobia*; *Methanosarcinales*; *Methanosaetaceae*; *Methanocellales***				
**Genus: *Methanothrix***				
*Methanothrix thermophila*	AN	(55)	6.1–7.5 (6.7)	Mesophilic anaerobic sludge digestors
	COH			
***Archaea*; *Euryarchaeota*; *Methanomicrobia*; *Methanosarcinales*; *Methanosaetaceae*; *Methanocellales***				
**Genus: *Methanocella***				
*Methanocella conradii*	AN	37–60 (55)	6.4–7.2 (6.8)	Rice field soil
	CLA			

## 3. Metabolism and Biotechnological Applications

In Table 2 potential applications for some of the described species are reported, particularly for the production of bioactive molecules and/or biocatalysts that may be important for industrial processes and biotechnologies.

**Table 2:** Biotechnological applications of major groups of extremophiles.

Enzymes, organic compounds and processes	Applications and products	Most representing Genera
Amylases and pullulanases	Glucose, fructose for sweeteners; polymer-degrading additives in detergents	*Pyrococcus*, *Thermococcus*, *Fervidobacterium*, *Dictyoglomus*, *Anaerobranca*
Cellulases and Xylanases	Paper bleaching	*Clostridium*, *Petrotoga*, *Thermotoga*, *Thermosypho*, *Moorella*, *Caldicoprobacter*, *Caldicellulosiruptor*
Proteases	Amino acid production from keratins, food processing, baking, brewing, detergents	*Thermoanaerobacter*, *Fervidobacterium*
DNA-polymerases and ligases	Genetic engineering	*Thermotoga*, *Pyrococcus*, *Thermococcus*, *Archaeoglobus*, *Thermoanaerobacter*
Ethanol	Chemical and food industries	*Clostridium*, *Thermoanaerobacter*; *Thermoanaerobacterium*, *Caldanaerobius*, *Caloramator*
Hydrogen and/or methane	Energy, fuels	*Clostridium*, *Carboxydocella*, *Thermincola*, *Thermosinus*, *Thermotoga*, *Carboxydothermus*, *Carboxydobrachium*, *Anaerobaculum*, *Methanotorris*, *Methanococcus*, *Methanothermococcus*, *Methanotermobacter*
Volatile fatty Acids	Chemical and food industries	*Clostridium*

The data reported here represent a summary of all that has been proposed and applied. A more exhaustive list of applications has been published by Vieille and Zeikus (2001).

Major metabolic possibilities can be observed in thermophiles, and there is no correlation between thermophily and metabolic properties, maybe with the exception of the reverse situation, *i.e.*, that the temperature limit for phototrophy is presently far below 70 °C. Amend and Shock [48] have previously described thermophilic and hyperthermophilic energenic reactions in depth, and their work is a key resource for the study of thermophilic metabolisms [49].

Chemoorganoheterotrophic metabolism (frequently in an incomplete form referred to as “heterotrophic”) can be further divided into subcategories according to the substrates and include glycolytic, (hemi)cellulolytic, lipolytic, and proteo/peptidolytic metabolisms, amongst others. The Emden-Meyerhof and Entner-Doudoroff pathways are employed by glycolytic thermophilic anaerobes, but a variety of modifications have been discovered, predominantly within the *Archaea* [50]. Major fermentation products formed by glycolytic thermophilic anaerobes include acetate, butyrate, lactate, ethanol, CO_2_, and H_2_ and to a lesser degree the observed products propionate, propanol and butanol. Traces of various branched fatty acids from amino acid degradation are also detected since many glycolytic anaerobic thermophiles require yeast extract for growth and some even for metabolic activity.

The production of ethanol by glycolytic and cellulolytic taxa has been studied. Cellulose and hemicellulose are the most abundant renewable natural plant fibers, and their degradation, coupled with the production of “biofuels”, such as ethanol by thermophilic anaerobes has been an intensely studied research area for the last 30 years, although research on fuel production leading to patents had already been done in the late 1920s, which includes the description and use of the oldest validly published anaerobic thermophile, *Clostridium thermocellum*. Recently, the focus has been shifting to butanol- and to H_2_-production. An example for this is the use of the *Caldicellulosiruptor bescii* strain DSM 6725^T^ [51] and of similar anaerobic thermophilic bacteria [52].

As with cellulose-degrading thermophilic anaerobes, xylanolytic thermophilic anaerobes generate interest because the conversion of xylan—a component of plant hemicellulose and the second-most abundant renewable polysaccharide in biomass—to useful products might be coupled with an increasing efficiency of processing lignocellulose and the production of energy from renewable resources. Xylan is widely used as carbon and energy source among thermophilic anaerobic *Bacteria*, especially among members of the Firmicutes [53,54,55,56].

Among chemolithoautotrophic pathways, the methanogenic reaction 4H_2_ + CO_2_ → CH_4_ + 2H_2_O, is well characterized and used by thermophilic taxa within the *Methanobacteriaceae*, *Methanothermaceae*, *Methanocaldococcaceae*, and *Methanococcaceae*. Another, relatively recently described, interesting chemolithoautotrophic metabolism of anaerobic thermophiles makes use of CO, which occurs as a normal component of escaping volcanic gas of terrestrial and deep-sea hydrothermal origin. Several thermophilic anaerobes that grow lithotrophically on CO have indeed been isolated, performing the metabolic reaction CO + H_2_O → CO_2_ + H_2_ employed by the acetogens *Desulfotomaculum*, *Carboxydothermus*, *Hermolithobacter*, *Carboxydocella*, *Thermincola*, *Caldanaerobacter*, and *Thermosinus* [57].

The same CO-using reaction has also been observed within the *Archaea* in an isolate belonging to the genus *Thermococcus* (family *Thermococcaceae*)*.* Another interesting chemolithotrophic strategy is employed by the acetogens using the Wood-Ljungdahl pathway (from the reaction: 3H_2_ + CO_2_ → acetate). Both mesophilic and thermophilic taxa (e.g., *Moorella* species) are known to perform this reaction.

Chemolithotrophs generate energy chemolithotrophically and assimilate carbon heterotrophically. Thermophilic anaerobes with this metabolism include *Archaea* as *Archaeoglobus profundus* and *Stetteria* *hydrogenophila*, and *Bacteria* as *Desulfotomaculum alkaliphilum*, *Desulfotomaculum carboxydivorans*, *Thermincola carboxydiphila*, *T. ferriacetica* (which can also grow hemolithoautotrophically), *Caldithrix abyssi*, *Vulcanithermus* *ediatlanticus*, and *Oceanithermus profundus*.

Two mechanisms for collecting light energy and converting it into chemical energy are known: one depends on photochemical reaction centers containing (bacterio)-chlorophyll and the other employs rhodopsins. However, to the authors’ knowledge, there are no rhodopsin-using thermophilic anaerobes yet described.

Within the phylum *Firmicutes* (family Heliobacteriaceae), *Heliobacterium modesticaldum* is an obligately anaerobic photoheterotroph that is also capable of growing chemoorganoheterotrophically [58]. *H.* *modesicaldum* is among the most recently discovered taxa containing (bacterio)-chlorophyll photochemical reaction centers; however, at present, it is not characterized in detail.

Many *Archaea* were initially described as being obligately dependent on S_0_ reduction for the production of energy, but it has often been reported that some of the so-called “sulfur-dependent” *Archaea* grow well in co-culture with hydrogen-using thermophilic methanogens in the absence of sulfur. This is possible through interspecies hydrogen transfer, whereby growth-inhibiting hydrogen (from H^+^ used as an electron acceptor) is removed without sulfur serving as the electron acceptor.

The *Ignicoccus–*“*Nanoarchaeum*” system has been described as a symbiotic relationship. It was discovered that small cocci were attached to the larger cells of a strain of *Ignococcus* isolated from the Kolbeinsey Ridge, in the north of Iceland [59]. These tiny cocci could be isolated from the larger cells and subsequently studied, but grew only when attached to their host. The genome sequence analysis of “*Nanoarchaeum*” showed that it was missing most of the enzymes required for nonparasitic growth.

The importance of sulfur in the metabolism of thermophilic anaerobes becomes evident when one considers that the majority of thermophiles (chemolithotrophs, as well as chemoheterotrophs) take advantage of the sulfur redox system. Amend and Shock [48] posed that the most common energy-yielding reaction under thermophilic conditions may be the reduction of elemental sulfur: H_2_ + S° → H_2_S.

Indeed, the diversity of known thermophilic anaerobic taxa that use this strategy is notable: the sulfur-reducing reaction has been reported within the *Pyrodictiaceae*, *Sulfolobaceae*, *Thermoanaerobacteriaceae*, *Thermoproteaceae*, *Aquificacea*, *Desulfurellaceae*, *Desulfurococcaceae*, *Thermococcaceae*, *Thermoplasmataceae*, *Thermofilaceae*, and *Thermotogaceae* genera. Thermophilic, sulfate-reducing *Bacteria* have been isolated from a wide range of environments, and many of these thermophiles belong to a phylogenetically coherent cluster of spore-forming *Desulfotomaculum* species (*Peptococcaceae* in the Phylum *Firmicutes*).

Thus, the role of sulfur in the metabolisms of thermophilic anaerobes can vary for different groups: it can be reduced, it can serve as an electron sink during fermentation, and it can function as a terminal electron acceptor to allow sulfur respiration.

Thermophilic anaerobic Fe(III)-reducing *Bacteria* and *Archaea* are found within nearly all thermobiotic environments and are usually diverse in terms of respiration, capable of growing chemoorganotrophically with fermentable substrates or chemolithoautotrophically with molecular hydrogen. Although only relatively recently described, a diverse set of thermophilic anaerobes is known to reduce Fe(III) [60]. Families of the *Bacteria* with taxa known to reduce Fe(III) include the *Bacillaceae*, *Peptococcaceae*, *Thermoanaerobacteriaceae*, *Acidaminococcaceae*, *Syntrophomonadaceae*, *Deferribacteraceae*, *Hydrogenothermaceae*, *Thermotogaceae*, and the *Thermodesulfobacteriaceae*. Families of the *Archaea* with taxa known to reduce Fe(III) include the *Thermoproteaceae*, *Archaeoglobaceae*, and the *Thermococcaceae.* *Geoglobus ahangari*, of the *Archaeoglobaceae,* was reported as the first dissimilatory Fe(III)-reducing prokaryote obligately growing autotrophically on hydrogen. In some genera, such as *Thermoanaerobacter*, *Thermotoga*, and *Anaerobranca*, many of the species tested have been found to be capable of dissimilatory reduction of Fe(III), but overall it appears as though the ability to reduce Fe(III) does not correlate with an affiliation at the genus or species level. For example, although *Deferribacter* *abyssi* and *Deferribacter thermophilus* are closely related, having 98.1% 16S rRNA gene sequence similarity, *D. abyssi* is unable to reduce Fe(III) whereas it is a primary electron acceptor for *D. thermophilus.* The chemolithoautotrophic iron reducers are of special interest since they are believed to have been responsible for the Low Temperature Banded Iron Formations. Beside the dissimilatory iron reduction, several thermophiles are also able to use various other metals, sometimes in combination with iron, sometimes they only reduce other oxidized metal ions, either as soluble ions or even within specific minerals. *Pyrobaculum arsenaticum* has the ability to grow chemolithotrophically by arsenate reduction, and both *P. arsenaticum* and *Pyrobaculum aerophilum* can use selenate, selenite, or arsenate chemolithoorganotrophically. For some thermophiles it appears that the reduction of metal ions occurs partly or fully without energy formation through this process as a detoxification mechanism. *Thermoanaerobacter* strains isolated from the Piceance Basin in Colorado were able to reduce Co(III), Cr(VI), and U(VI), in addition to Mn(IV) and Fe(III) [61].

In addition to these described characteristics—O_2_-relationship, temperature and pH profiles, and metabolic strategies—a number of additional physiological properties of thermophilic anaerobes should be examined and should, therefore, add to what is known about the diversity of thermophilic anaerobes. The NaCl optimum and tolerance of a prokaryote is often assessed. Thermophilic anaerobes of marine origin, for example, would be expected to grow best at marine salinity—around 3.5% (wt/vol) NaCl. Prokaryotes that grow optimally with high salinity are referred to as halophiles, and halophilic thermophilic anaerobes are known, as are halophilic alkalithermophiles [62].

Thermophilic anaerobes living at deep-sea hydrothermal vent sites must cope with the additional pressure exerted by the water column and are, therefore, piezotolerant or perhaps even piezophilic [63,64]. Both *Methanocaldococcus* (basonym *Methanococcus*) *jannaschii,* isolated from the 21 °N East Pacific Rise deep-sea hydrothermal vent site, and *Thermococcus barophilus*, obtained from the Snakepit region of the Mid-Atlantic Ridge, grow faster under increased hydrostatic pressure [22,23].

At its optimal growth temperature, the growth rate of *T. barophilus* was more than doubled at elevated hydrostatic pressure (40 MPa) compared with the growth rate at low pressure (0.3 MPa). Furthermore, *T. barophilus*, as well as “*Pyrococcus abyssi*” and *Pyrococcus* strain ES4, isolated from deep-sea hydrothermal vent sites, show an extension of their T_max_ with significant elevated hydrostatic pressure [21,22,23].

Representative genera of thermophilic anaerobes living at deep-sea hydrothermal vent sites include *Archaeoglobus*, *Thermodiscus*, *Thermoproteus*, *Acidianus*, *Pyrococcus*, *Thermococcus* and *Desulfurococcus*, which reduce sulfur or sulfate, *Sulfolobus* can oxidize H_2_S or elemental sulfur, the methanogens *Methanothermus*, *Methanococcus* and *Methanopyrus*, and the nitrate reducers *Pyrobaculum* and *Pyrolobus*. *Sulfolobus* and *Acidianus* isolates can also oxidize ferrous iron, and with no doubt such a process plays a major role on the local environment and biogeochemical cycles. Examples of hyperthermophilic bacteria are included in the genera *Thermotoga* and *Aquifex*.

Some of the isolated thermophilic anaerobes also possess ionizing radiation resistance; for example, this characteristic is found in *Tepidimicrobium ferriphilum* (Order *Clostridiales*), which was isolated from a freshwater hot spring within the Barguzin Valley, Buryatiya, Russia [65]. The level of natural radioactivity at hydrothermal vents can be 100 times greater than that at Earth’s surface because of the increased occurrence of elements, such as ^210^Pb, ^210^Po and ^222^Rn. Indeed, *Archaea* of the family *Thermococcaceae*, *Thermococcus gammatolerans* and *Thermococcus* “*radiotolerans*” isolated from the Guaymas Basin, of the Gulf of California, and *Thermococcus* “*marinus*”, isolated from the Snakepit hydrothermal site of the Mid-Atlantic Ridge have γ-irradiation resistance.

It is worth mentioning the moderate thermophiles and thermotolerant organisms, particularly for their potential applications as well as for their ecological roles. Among these are the cellulolytic *Clostridium thermocellum*, the acetogenic *Moorella thermoacetica/thermoautotrophica* and *Thermoanaerobacterium (former Clostridium) thermosaccharolyticum*, capable of growing in vacuum packed foods and thus known as the “can-swelling” organism [66,67,68,69]. The obligate mixotrophic *Thiomonas bhubaneswarensis*, the marine *Lutaonella thermophila* and *Thermophagus xiamenensis*, the cellulolytic bacteria *Clostridium clariflavum* and *Clostridium caenicola*, the faculatative microaerophilic *Caldinitratiruptor microaerophilus*, and a novel hydrogen-producing bacterium from buffalo-dung were described [70,71,72,73,74,75].

Novel isolates were isolated from waste disposal plants, methanogenic reactors and wetland systems. *Tepidanaerobacter acetatoxydans*, *Anaerosphaera aminiphila* and *Clostridium sufflavum* were isolated from two methanogenic processes [76,77,78], whereas *Anaerosalibacter bizertensis* and *Gracilibacter thermotolerans* were observed and described in artificial ecosystems [79,80].

## 4. Conclusions

Anaerobic thermophilic microorganisms have been known for a long time but it is always difficult to understand that some organisms do not only survive at high temperatures, but actually thrive in boiling water. They are one of the most interesting varieties of extremophilic organisms.

The main interest in anaerobic thermophiles during the last decades has mainly been on two issues dealing with basic and applied research: 1) the discovery of many novel hyperthermophilic *Archaea* (of which many can grow at 100 °C and above and a few even up to 121 ^o^C), has attracted a great interest among the scientific community; 2) the realization that anaerobic thermophilic microorganisms can serve as excellent sources for thermostable biocatalysts was the driving force for implementing basic and applied research on thermophiles.

Due to the stress of living at such extreme temperatures, anaerobic thermophiles have evolved a variety of mechanisms that allow them to survive at temperatures other organisms cannot thrive at. These traits include unique membrane lipid composition, thermostable membrane proteins, and higher turnover rates for various protein enzymes. One of the most important attributes to the maintenance of homeostasis within the organism is that of the plasma membrane surrounding the organism. Aside from having to stabilize the plasma membrane at high temperatures, anaerobic thermophiles must also stabilize their proteins, DNA, RNA, and ATP. Study into how they manage thermostability at the protein and membrane structural level has elucidated many traits of protein, membrane and nucleic acid structure; however, there is not yet a full understanding of the principles of thermophily and thermostability of cell components. As a matter of fact, the process of heat stabilization for DNA, RNA, and ATP is not fully understood yet.

With no doubts anaerobic thermophiles are interesting from the viewpoint of the trend toward biotechnology as many chemical industrial processes employ high temperatures which would have to be lowered in order to use bioprocesses from mesophiles, and this could be avoided using enzymes of thermophiles.

One of the most interesting potential application of anaerobic thermophilic microorganisms is the production of biofuels that was particularly investigated in the last decades, mainly as research activities on the metabolism of pure or mixed cultures to produce biofuel, including methane and hydrogen, but also throughout extensive lab work with the aim to obtain ethanol from biomass by means of thermophilic biological processes.
